# An in vitro cytotoxicity of glufosfamide in HepG2 cells relative to its nonconjugated counterpart

**DOI:** 10.1186/s43046-021-00080-6

**Published:** 2021-08-23

**Authors:** Doaa E. Ahmed, Fatma B. Rashidi, Heba K. Abdelhakim, Amr S. Mohamed, Hossam M. M. Arafa

**Affiliations:** 1 Department of Biochemistry, Misr University for Science & Technology (MUST), Giza, Egypt; 2 Biochemistry Lab.Department of Chemistry, Faculty of Science, Cairo University, Giza, Egypt; 3 Department of Pharmacology and Toxicology, Faculty of Pharmacy Ahram Canadian University, Giza, 267119 Egypt

**Keywords:** Glufosfamide HepG2 cytotoxicity, Ifofosfamide (IFO), Bcl-2, Caspase-9, Mitochondrial membrane potential

## Abstract

**Background:**

Glufosfamide (β-d-glucosylisophosphoramide mustard, GLU) is an alkylating cytotoxic agent in which ifosforamide mustard (IPM) is glycosidically linked to the β-d-glucose molecule. GLU exerted its cytotoxic effect as a targeted chemotherapy. Although, its cytotoxic efficacy in a number of cell lines, there were no experimental or clinical data available on the oncolytic effect of oxazaphosphorine drugs in hepatocellular carcinoma. Therefore, the main objective of the current study is to assess the cytotoxic potential of GLU for the first time in the hepatocellular carcinoma HepG2 cell line model.

**Methods:**

Cytotoxicity was assayed by the MTT method, and half-maximal inhibitory concentration (IC_50_) was calculated. Flow cytometric analysis of apoptosis frequencies was measured by using Annexin V/PI double stain, an immunocytochemical assay of caspase-9, visualization of caspase-3, and Bcl2 gene expression were undertaken as apoptotic markers. Mitochondrial membrane potential was measured using the potentiometric dye; JC-1, as a clue for early apoptosis as well as ATP production, was measured by the luciferase-chemiluminescence assay.

**Results:**

Glufosfamide induced cytotoxicity in HepG2 cells in a concentration- and time-dependent manner. The IC_50_ values for glufosfamide were significantly lower compared to ifosfamide. The frequency of apoptosis was much higher for glufosfamide than that of ifosfamide. The contents of caspase-9 and caspase-3 were elevated following exposure to GLU more than IFO. The anti-apoptotic Bcl2 gene expression, the mitochondrial membrane potential, and the cellular ATP levels were significantly decreased than in case of ifosfamide.

**Conclusions:**

The current study reported for the first time cytotoxicity activity of glufosfamide in HepG2 cells in vitro. The obtained results confirmed the higher oncolytic activity of glufosfamide than its aglycone ifosfamide. The generated data warrants further elucidations by in vivo study.

## Background

Hepatocellular carcinoma (HCC) is a malignant tumor that arises from hepatocytes, the major cell type in the liver. It is the most common primary hepatic tumor and the fifth most common tumor worldwide. It has a high incidence in sub-Saharan Africa and Asia. About 46,000 new cases of hepatocellular carcinoma have been recorded to be diagnosed in sub-Saharan Africa each year, and age-standardized incidences of the tumor as high as 41.2/100,000 persons/year [1]. At the molecular level, HCC is a heterogeneous disease. It occurs in more than 80% of the cirrhotic liver, but the molecular pathways involved depend on the cause of cirrhosis. Liver carcinogenesis may last for decades, through progressive accumulation of different genetic alterations that eventually lead to malignant transformation. Thus, chronic liver injury initiates increased liver cell turnover, triggering oxidative DNA damage and inflammatory events. This leads to the formation of dysplastic and macro-regenerative nodules which are considered to be neoplastic nodules [2].

Systemic chemotherapy lacks a radical cure for HCC, and there is currently no standard treatment for patients with non-resectable HCC. Local ablation has shown strategies such as ethanol injection, radiofrequency, and cryotherapy can prolong survival in selected patients and prevent tumor progression while on a waiting list for transplantation. Innovative approaches such as targeting the non-transformed, less resistant, tumor-supporting endothelial cells may change this outcome [3].

Glufosfamide, a nitrogen mustard derivative, is an analog of oxazaphosphorine drugs. These belong to a class of bifunctional alkylating agents of potential chemotherapeutic activities towards varieties of human malignancies. Several new analogs of these drugs family including glufosfamide have been designed to increase therapeutic selectivity and reduce off-target toxicity, in comparison with their ascendants [4]. Glufosfamide (D-19575, β-d-glucose-isophosphoramide mustard, ß-D-Glc-IPM) is the next-generation glucose conjugate of ifosfamide; is a glucose conjugate of ifosfamide, in which isophosphoramide mustard, the bioactive alkylating metabolite of ifosfamide; and is covalently linked to β-d-glucose [5]. Glufosfamide has the potential to target tumor cells by serving as a substrate for such glucose transporters in the plasma membrane [6]. Together with the increased metabolic rate and glucose consumption of tumor cells, this targeting mechanism may contribute to the relative selectivity of the drug for tumor cells.

Another characteristic of glufosfamide is that, because of the absence of the oxazophosphorine ring in its structure, it does not release the urothelium irritant acrolein, which has been shown to induce hemorrhagic cystitis in individuals treated with IFO. Moreover, the amount of toxic chloroacetaldehyde generated metabolically after administration of D-19575 is markedly reduced compared with that generated after IFO administration. Preclinical pharmacokinetic analysis has revealed that glufosfamide is rapidly cleared by the kidney and has favorable tissue distribution and protein-binding profiles [7].

Although glufosfamide can trigger three forms of programmed cell death, namely, apoptosis, autophagy, and necrosis, the mechanisms of the action of glufosfamide have not yet been fully elucidated. Indeed, glufosfamide has proven apparent cytotoxicity in an array of tumor cell lines [4], and there was no data are available, however, on its oncolytic effect in hepatocellular carcinoma whether experimental or clinical.

Therefore, the objective of the current study aimed to assess and compare the cytotoxic potential of glufosfamide and its parent aglycone ifosfamide in a hepatocellular carcinoma cell line paradigm, namely, HepG2, in addition to shedding more light about the molecular mechanisms underlying the possible cytotoxic potential of both oxazophosphorine drugs.

## Methods

### Chemicals and drugs

GLU was obtained from Threshold Pharmaceuticals (South San Francisco, USA). Ifosfamide was obtained from Baxter Oncology GmbH (Weiterstadt, Germany). Dulbecco’s modified Eagle’s medium (DMEM): It was purchased from Life Technologies (CA. USA) and was used as a basal medium for supporting the growth of HepG2 cells. Fetal bovine serum (FBS): Sterile solution (10%) was obtained from Sigma-Aldrich (St. Louis, MO, USA). l-Glutamine (200 mM) was obtained from Sigma-Aldrich (St. Louis, MO, USA). MTT (1–3(4, 5-dimethylthiazol-2-yl)-2,5-diphenyltetrazolium bromide) was prepared as 0.5 mg/ml solution and used for the evaluation of cytotoxicity. Trypan blue dye was obtained from Sigma-Aldrich (St. Louis, MO, USA). The dye was used to stain dead cells during the assessment of cytotoxicity. Trypsin/EDTA was purchased from Lonza Walkersville (Washington, USA). It was supplied as a solution of trypsin (200 mg/L) and EDTA (17,000 U/L). It was used in a concentration of 0.25% (w/v) Trypsin/0.53 mM EDTA to detach HepG2 cells. Penicillin (10,000 U/mL)/streptomycin (10 mg/mL) was obtained from Sigma-Aldrich (St. Louis, MO, USA) and used for sterilization of cell cultures. ANNEXIN V FITC/Pl apoptosis detection kit was purchased from Beckman Coulter GmbH (Krefeld, Germany) and used for flow cytometric detection of apoptosis. SYBR® Green PCR Master Mix and SYBR® Green RT-PCR Reagents Kit were purchased from Applied Biosystems (Life Technologies, CA, USA) and used to perform real-time PCR and direct detection and monitoring of PCR product. JC-1 mitochondrial membrane potential assay kit was purchased from Cayman Chemical Company (MI, USA) and used for the assessment of mitochondrial membrane potential in cultured HeG2 cells. Cell Titer-Glo Luminescent Cell Viability Assay kit, this kit was commercially obtained from Promega (CA, USA) and used for the cellular determination of ATP levels in HepG2 cells.

### Cell culture

HepG2 (hepatocellular carcinoma cell line, ATCC Number HB-8065) was purchased from the Holding Company for Biological Products & Vaccines (VACSERA, Agouza, Giza, Egypt). Cells were cultured in tissue culture flasks (75-cm2 surface area) using RPMI supplemented with 10% fetal bovine serum (FBS) as a growth culture medium and glutamine (200 mM). Preliminary cultures and passages were made in the DMEM medium. The cell line was maintained according to the method earlier described by [8]. After discarding the culture medium, the cell layer was gently washed with sterile phosphate buffer saline (PBS), the cell layer was rinsed with 0.25% (w/v) Trypsin- 0.53 mM EDTA solution to remove all traces of serum that contain trypsin inhibitor. About 2–3 ml of trypsin–EDTA solution was added to the flask and cells were observed under an inverted microscope until the cell layer was dispersed (usually within 5 to 15 min). Cells that are difficult to detach may be placed at 37 °C in CO_2_ incubator to facilitate dispersal. Six to 8 ml of complete growth medium was added to the cells to inhibit trypsin and then incubated in 5% CO_2_ incubator at 37 °C. The medium renewal was done twice per week. Cell counting and viability check were performed using Dye Exclusion Technique. A cell number was calculated by counting the cells using a hemocytometer according to the method described by [9].

### Cytotoxicity assay

Cytotoxicity of both glufosfamide and ifofosfamide was evaluated in cultured HepG2 cells. Experiments were designed to elucidate the cytotoxic potencies of both oxazophosphorine congeners on different time periods. Stock solutions were prepared by dissolving either drug in free medium. Drug concentration–response curves were assessed. Seeding was done at a density of 5000 cells/well in 96-well plates. Drug were used at concentrations of 10–1000 μM. After cell conditioning and maintenance (as described in the “Material and methods” section), cells were treated with drug concentrations and incubated for different time intervals; 24, 48, and 72 h. Cytotoxicity was assessed at the end of drug exposure using MTT assay as earlier described by Mosmann [10]. Following 24 h of cell plate incubation, the medium was removed and 200 μl of medium containing different concentrations of either glufosfamide or ifosfamide (10–1000 μM) were added. After varying times of incubation the medium was removed, and for staining, 100 μl of MTT solution in PBS was added to each well at final concentration of 0.5 mg/ml medium and incubated 1–5 h. Then, media were dumped off and the plate were dried on paper towels, and formazan (MTT metabolic product) was made soluble in 200 µl DMSO for 5 min on a shaker at 150 rpm. Absorbance was then measured using an ELISA plate reader at a test wavelength of 570 nm and a reference wavelength of 630 nm to obtain sample signal. Optical density should be directly correlated with cell quantity. Results were expressed as the relative percentage of absorbance compared to control. The cytotoxicity of each concentration was tested in triplicate and, and each experiment was repeated once to make an overall sample size of *n* = 6. The half maximal inhibitory concentration (IC_50_) was calculated using Graphpad Prism 5 software (San Diego, CA, USA). Drug interactions were analyzed by Calcusyn program version 2.1 (Biosoft, Cambridge, UK) based on the analytical method of Chou and Talalay [11].

### ANNEXIN V FITC/Pl assessment of apoptosis

Apoptosis was performed using ANNEXIN V FITC/Pl apoptosis assay kit (Krefeld, Germany) to quantify the number of cells undergoing apoptosis following tagging with annexin v protein. The tagged cells were visualized by flow cytometer. The apoptotic effects of the IC_50_ values (and some fractions) of both glufosfamide and ifofosfamide were addressed in the current study. After HepG2 cells conditioning and maintenance as in the “Materials and methods” Sect. (2.2.), they were treated with the test drug concentrations. The optimal incubation time before pulsation of the cells into the flowcytometer was selected from the cytotoxicity study to be 72 h. Each experiment was done in triplicate and repeated once to give a sample size of *n* = 6. The percentage of apoptosis (early and late) was calculated, and the mean values for each drug were computed.

### Human caspase-9 measurement

Apoptosis was further confirmed by the assessment of the content of human caspase-9 in HepG2 cell lysates by ELISA kit obtained from Biovendor R & D (Heidelberg, Germany) as per manufacturer instruction. After cell conditioning and maintenance, cells were lysed by lysis buffer and subjected to anti-human caspase-9 antibody. Then, IC_50_ values of both glufosfamide and ifosfamide (fractions) were tested for their effects on human caspase-9 content. Experiments were done in triplicate and repeated one to a given sample size of *n* = 6.

### Bcl2 and caspase-3 gene expression measurement

HepG2 cells were prepared as mentioned above in the “Materials and methods” Sect. (2.2.), then the total RNA was extracted from cell lysate using SV total RNA isolation and purification system (Promega WI, USA), followed by reverse transcription. Then, the qPCR were carried out using gene-specific primers for caspase-3, Bcl2, and Beta actin, respectively, Fwd: 5’GTG TCT GGT CAT TTC CGA CTG A 3’, Rev: 5’ATA CTC CAC AGC ACC TGG TTA T 3’ according to gene bank accession number: NM004346. Fwd: 5’GCA GGC GAC GAG TTT GAA CT 3’ Rev: 5’GTG TCT GGT CAT TTC CGA CTG A 3’ according to gene bank accession number: NM138578 and Forward 5’ TCT GGC ACC ACA CCT TCT ACA ATG 3’ Reverse 5’ AGC ACA GCC TGG ATA GCA ACG 3’ accession number: XM_035085394.1. After calculation of the relative expression, IC_50_ values for both glufosfamide and ifosfamide were tested for the quantitative assessment of Bcl2 and caspase-3 genes using real time PCR. Each experiment was done in triplicate and repeated once to a given sample size of *n* = 6.

### Determination of the mitochondrial membrane potential in HepG2 cells

This experiment was conducted to unravel the effects of both glufosfamide and ifosfamide on the mitochondrial membrane potential of HepG2 cells as a clue for early apoptosis. This was done using a potentiometric dye, JC-1, which is a membrane permeable dye widely used for determining mitochondrial membrane potential using fluorometer, flowcytometry, and fluorescent microscopy [12]. Mitochondrial membrane potential, Δψm, is an important parameter of mitochondrial function used as an indicator of cell health. IC_50_ values obtained from the cytotoxicity study were used. Each experiment was done in triplicate and repeated once for a given sample size of *n* = 6. Mitochondrial membrane potential was a function of the difference in excitation between the red J-aggregates and the green monomeric dye.

### ATP concentration measurement in HepG2 cells

This test was performed as a mechanistic study to elucidate the effects of IC_50_ doses of glufosfamide and ifosfamide on cellular content of ATP using the luciferase-luminescence assay according to the method described by Mikirova et al. [13] using a commercial Cell Titer-GloLuminescent cell Viability Assay kit (Promega, USA). Luminescence relative units (LRU) were determined for each drug as count per seconds (CPS). Each dose was tested in triplicate and repeated once for a given sample size of *n* = 6. Correlation analysis was further done to disclose the possible relationship between disruption of mitochondrial membrane potential and energy production.

### Statistical analysis

Data are presented as mean ± SD; comparisons were carried out using one way analysis of variance (ANOVA) followed by Tukey–Kramer’s test for post hoc analysis. Statistical significance was acceptable to a level of *P* < 0.001. All statistical analysis was performed using Graph pad Prism 5 software (San Diego, CA, USA).

## Results

### Effects of glufosfamide or ifosfamide on the viability of HepG2 cells

**Fig. 1:**
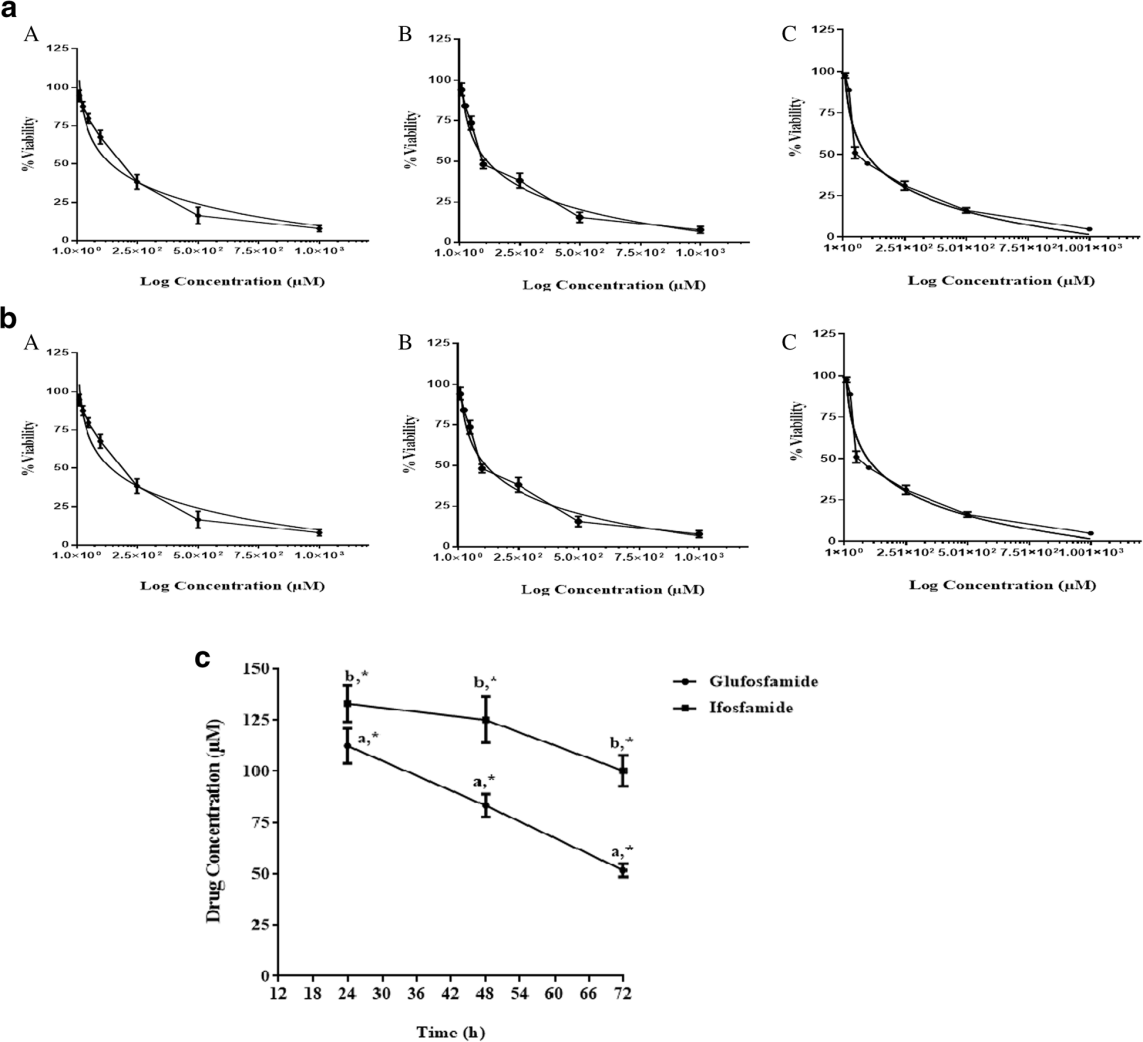
Concentration response curves and Mean IC50 values of glufosfamide and ifosfamide on HepG2 cells. **a** Concentration response curves testing the effect of glufosfamide on HepG2 cells at different times **A** 24 h, **B** 48 h, and **C** 72 h. **b** Concentration response curves testing the effect of ifosfamide on HepG2 cells at different times **A** 24 h, **B** 48 h, and **C** 72 h. **c** Mean IC50 values for glufosfamide and ifosfamide at various time intervals

To investigate the cytotoxic effect of glufosfamide or ifosfamide on HepG2, cell concentration response curves for each drug were assessed and compared. Both glufosfamide and ifosfamide showed enhanced cytotoxicity in HepG2 cells in a concentration- and time-dependent manner. The half-maximal inhibitory concentrations (IC_50_) of glufosfamide were 112.32 ± 8.5, 83.23 ± 5.6, and 51.66 ± 3.2 following the corresponding incubation periods; 24, 48, and 72 h, respectively (Table 1 and Fig. 1a). It was apparent that the optimal time period was at 72 h followed by 48 h and 24 h. The IC_50_ of ifosfamide were 133 ± 8.9, 125 ± 11.2, and 100.2 ± 7.6 following the respective incubation periods; 24, 48, and 72 h, respectively (Table 1 and Fig. 1b). The optimal time point was 72 h followed by 48 h and then 24 h. Moreover, the IC_50_ values for glufosfamide were significantly lower by about 16, 33, and 48% compared to ifosfamide at 24, 48, and 72 h, respectively (Table 1 and Fig. 1a–c).

**Table 1:** Mean IC_50_ values for glufosfamide or ifosfamide in HepG2 cells following different incubation time periods

**Incubation time**	**HepG2 Cells**	
	**Glufosfamide**	**Ifosfamide**
**24 h**	112.32^*,a^ ± 8.5	133.0^*,b^ ± 8.9
**48 h**	83.23^*,a^ ± 5.6	125.0^*,b^ ± 11.2
**72 h**	51.66^*,a^ ± 3.2	100.20^*,b^ ± 7.6

Data represent mean ± SE, *n* = 6 (Each concentration was processed in triplicate; each experiment was done twice)

IC50 values were in µM

Each drug was added to culture medium at concentration range; 10–1000 µM

HepG2 cells were seeded at 5000–10,000 cells per well

Cytotoxicity was assessed using MTT assay

^*^Data in rows having the same superscript are significantly different from each other at *P* < 0.05 using independent two-tailed Student’s *t *test

^a, b^Data in columns having the same superscript are significantly different from each other at *P* < 0.05 using one way analysis of variance (ANOVA) followed by Tukey as post hoc test

### Effects of glufosfamide or ifosfamide on the frequency of apoptosis following Annexin V FITC/Pl double stain

**Fig. 2:**
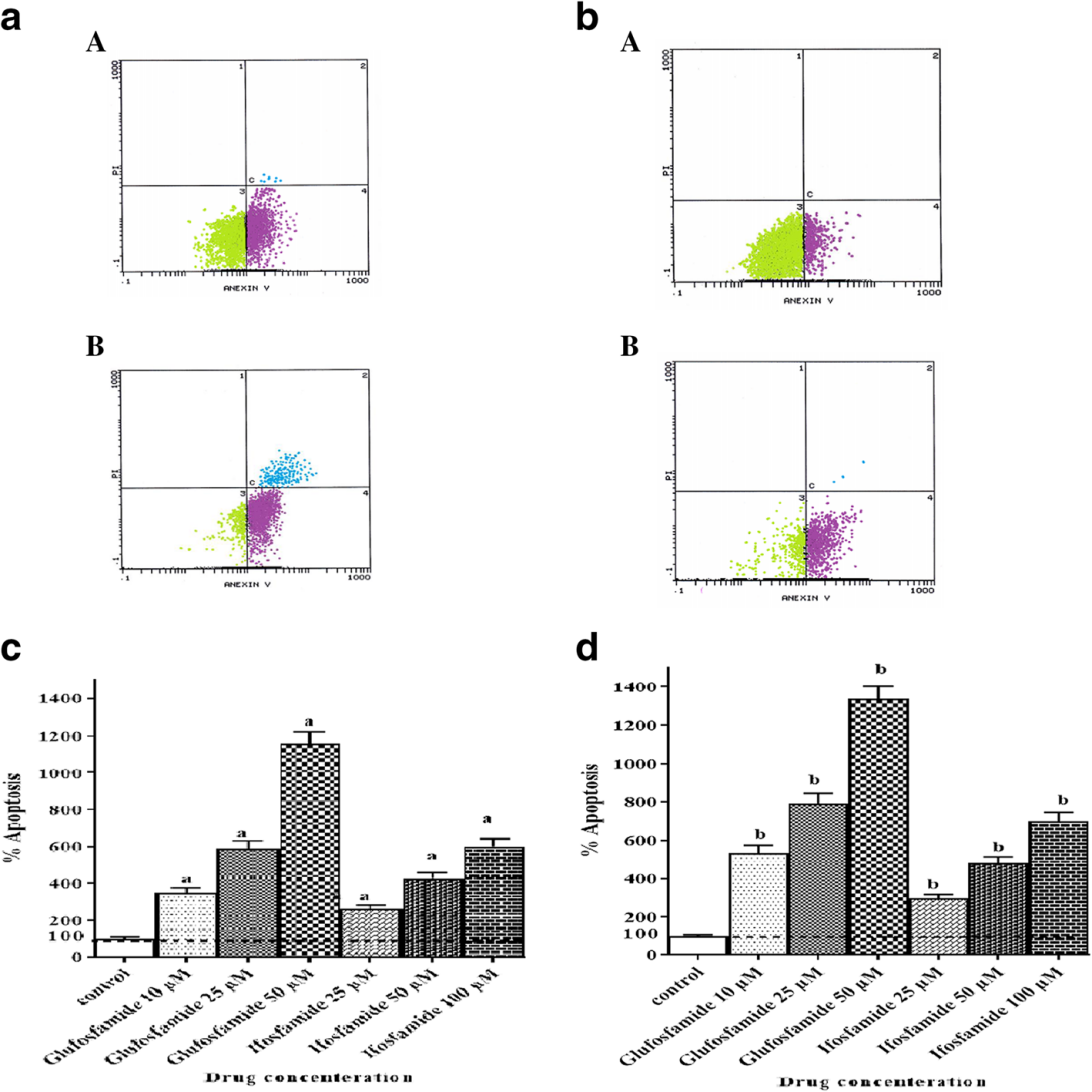
Effects of glufosfamide or ifosfamide on the frequency of apoptosis following Annexin V FITC/Pl double stain. **a** ANNEXIN V FITC/Pl apoptosis assay for HepG2 cells after glufosfamide IC_50_ (50 µM) treatment for **A** 48 h and **B** 72 h. **b** ANNEXIN V FITC/Pl apoptosis assay for HepG2 cells after ifosfamide IC50 (100 µM) treatment for **A** 48 h and **B** 72 h. **c** Comparative effects of glufosfamide or ifosfamide on the frequency of apoptosis in HepG2 cells following incubation for 48 h using Annexin V FITC/Pl double stain. **d** Comparative effects of glufosfamide or ifosfamide on the frequency of apoptosis in HepG2 cells following incubation for 72 h using Annexin V FITC/Pl double stain

Glufosfamide induced apoptosis as shown by the cells that stained positive for Annexin V FITC/Pl (Fig. 2a). The frequency of apoptosis following challenge of HepG2 cells with glufosfamide at concentrations; 10, 25, and 50 µM for 48 h increased by 252, 487, and 1058% respectively, compared to non-treated cells (Table 2 and Fig. 2c). Likewise, ifosfamide induced apparent positive staining of HepG2 cells following incubation for 48 h (Fig. 2b). Ifosfamide increased the percent apoptosis by about 165, 362, and 500% when used at concentrations; 25, 50, and 100 µM, respectively, compared to non-treated control cells (Table 3 and Fig. 2c). It was apparent that the frequency of apoptosis following incubation with HepG2 cells for 48 h was much higher in case of glufosfamide compared to ifosfamide. For instance, the IC_50_ of glufosfamide (50 µM) had higher apoptosis frequency amounted to 93% compared to the corresponding IC_50_ of ifosfamide (100 µM) (Fig. 2c). Furthermore, glufosfamide increased the incidence of apoptosis by 434, 689, and 1237% following incubation with HepG2 cells for 72 h at concentrations; 10, 25, and 50 µM, respectively, compared to non-treated cells (Table 2 and Fig. 2d). Ifosfamide also increased apoptosis frequency in HepG2 cells when incubated for 72 h at concentrations 25, 50, and 100 µM by 197, 380, and 600%, respectively, compared to non-treated control cells (Table 3 and Fig. 2d). Moreover, the incidence of apoptosis following incubation with HepG2 cells for 72 h was much higher in case of glufosfamide compared to ifosfamide. For instance, the IC_50_ of glufosfamide (50 µM) provoked higher apoptosis frequency amounted to 91% compared to the corresponding IC_50_ of ifosfamide (100 µM) (Fig. 2d). Interestingly, incubation of glufosfamide with HepG2 cells for 72 h exhibited higher apoptosis frequencies than those observed after 48 h post-incubation at all concentrations. For instance, the percent apoptosis at 72 h post-incubation was significantly increased by 72, 52, and 30% for the concentrations 10, 25, and 50 µM, respectively, compared the 48-h incubation period for the same concentrations (Table 2). By the same taken, ifosfamide had higher apoptosis frequencies amounted to 27, 27, and 32% when incubated with the concentrations 25, 50, and 100 µM for 72 h compared to the percent of apoptosis observed following exposure of HepG2 cells to the same concentrations for only 48 h (Table 3).

**Table 2:** Effects of glufosfamide on the frequency of apoptosis in HepG2 cells

Time period	Control	*Glufosfamide (µM)*^*1*^		
		10	25	50
48 h	3.1 ± 0.1^a^	10.9^a^ ± 0.9	18.2^a^ ± 1.2	35.9^a^ ± 2.1
72 h	3.5 ± 0.9^b^	18.7^*,b^ ± 1.5	27.6^*,b^ ± 1.7	46.8^*,b^ ± 2.5

Data represent mean ± SE, *n* = 3

^1^Glufosfamide was used in its IC50 value beside two of its fractions; 10 and 25 µM

The number of HepG2 cells used was in the range of 5 × 105 ~ 5 × 106 cells/mL

Percent apoptosis was calculated as the sum of frequencies of cells in late apoptosis and those undergoing apoptosis

^*^Significantly different from the same concentration incubated with HepG2 cells for 48 h at *P* < 0.05, using independent two-tailed Student’s *t *test

^a,b^Data in the same rows having the same superscript are significantly different from each other at *P* < 0.05 using one-way analysis of variance (ANOVA) followed by Tukey as post hoc test

**Table 3:** Effects of ifosfamide on the frequency of apoptosis in HepG2 cells

***Time period***	***Control***	***Ifosfamide (µM)***^***1***^		
		**25**	**50**	**100**
**48 h**	3.1 ± 0.1^a^	8.2^a^ ± 0.9	13.2^a^ ± 1.1	18.6^a^ ± 1.5
**72 h**	3.5 ± 0.9^b^	10.4^*,b^ ± 0.6	16.8^*,b^ ± 1.3	24.5^*,b^ ± 2.1

Data represent mean ± SE, *n* = 3

1Ifosfamide was used in its IC50 value beside two of its fractions; 25 and 50 µM

The number of HepG2 cells used was in the range of 5 × 10^5^ ~ 5 × 10^6^ cells/mL

Percent apoptosis was calculated as the sum of frequencies of cells in late apoptosis and those undergoing apoptosis

^*^Significantly different from the same concentration incubated with HepG2 cells for 48 h at *P* < 0.05, using independent Student’s *t *test

^a,b^Data in the same rows having the same superscript are significantly different from each other at *P* < 0.05 using one-way analysis of variance (ANOVA) followed by Tukey as post hoc test

### Comparative effects of glufosfamide or ifosfamide on caspase-9 content in HepG2 cells

**Fig. 3:**
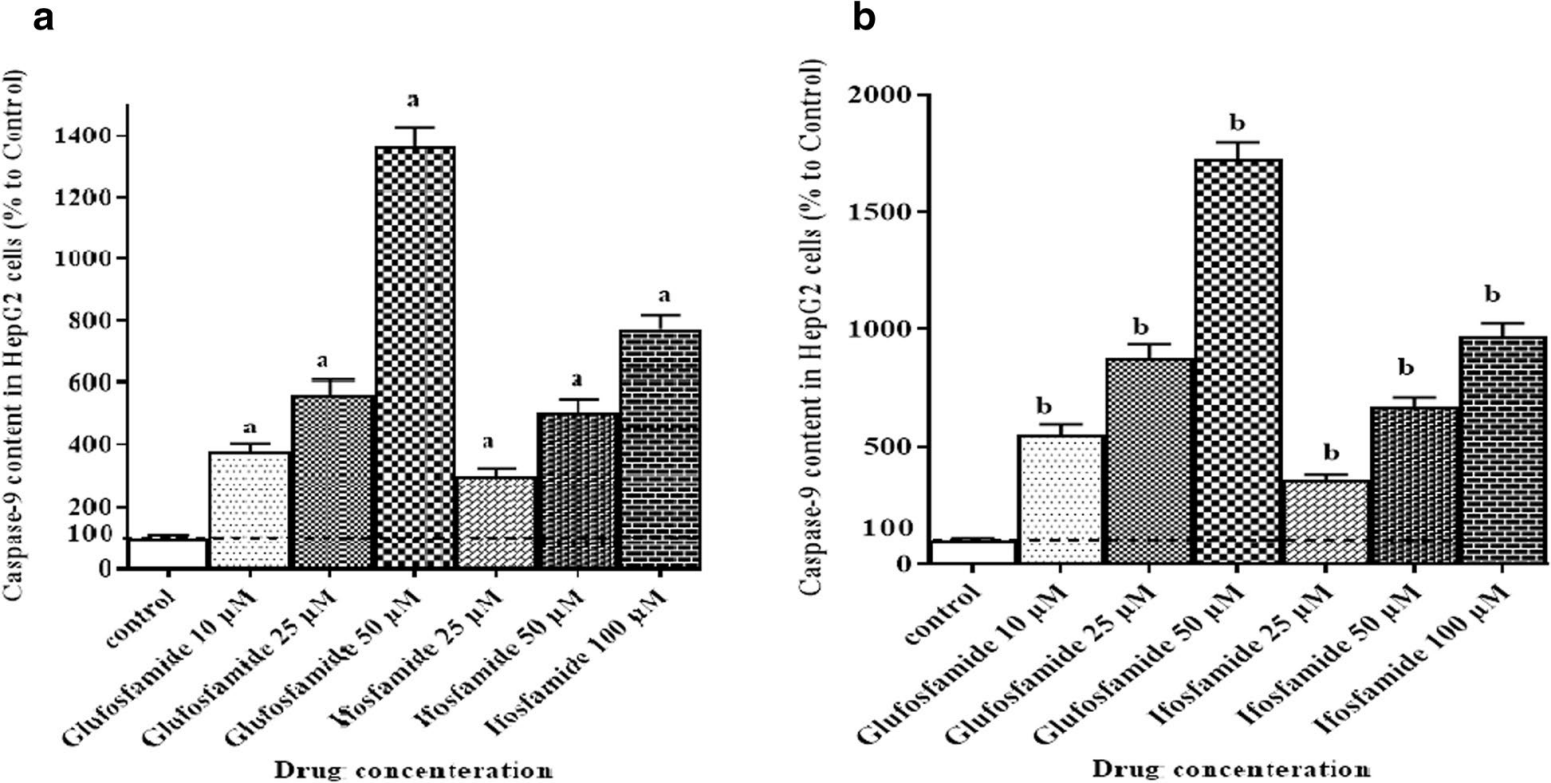
Effects of glufosfamide or ifosfamide on caspase-9 content in HepG2 cells. **a** Effects of glufosfamide and ifosfamide on caspase-9 content in the cell lysate of HepG2 cells at 48 h post-incubation. **b** Effects of glufosfamide or ifosfamide on caspase-9 content in the cell lysate of HepG2 cells at 72 h post-incubation

Glufosfamide increased caspase-9 content in HepG2 cells by 278, 463, and 1263% following incubation with cells for 48 h at concentrations; 10, 25, and 50 µM, respectively, compared to non-treated cells (Table 4 and Fig. 3a). Similarly, ifosfamide significantly increased the cellular content of the metalloproteinase by 203, 408, and 673% at 48 h post-incubation with 25, 50, and 100 µM concentrations, respectively, compared to non-treated control cells (Table 5 and Fig. 3a). Following incubation with HepG2 cells at the concentrations 10, 25, and 50 µM of glufosfamide for 72 h, there were increases in the cellular contents of caspase-9 amounted to 451, 733, and 1626% compared to non-challenged control cells (Table 4 and Fig. 3b). Likewise, ifosfamide increased caspase-9 content in HepG2 cells by 257, 569, and 870% following incubation with the drug at concentrations 25, 50, and 100 µM for 72 h, respectively, compared to non-treated cells (Table 5 and Fig. 3b). The cellular caspase-9 levels were significantly higher by 53, 56, and 33% following incubation of glufosfamide at concentrations; 10, 25, and 50 µM for 72 h, respectively, compared to the corresponding values following incubation for only 48 h (Table 4). Ifosfamide also increased caspase-9 content by about 24, 38, and 32% when incubated for 72 h at concentrations; 25, 50, and 100 µM, respectively, compared to those values obtained with the same concentration regimens at 48 h post-incubation (Table 5).

**Table 4:** Effects of glufosfamide on caspase-9 content in cell lysate of cultured HepG2 cells

***Time period***	***Control***	***Glufosfamide (µM)1***		
		**10**	**25**	**50**
**48 h**	4.0 ± 0.2^a^	15.1^a^ ± 1.3	22.5^a^ ± 1.2	54.5^a^ ± 2.5
**72 h**	4.2 ± 0.5^b^	23.13^*,b^ ± 1.9	35.0^*,b^ ± 2.3	72.5^*,b^ ± 4.1

Data represent mean ± SE, *n* = 3

^1^Glufosfamide was used in its IC_50_ value beside two of its fractions; 10 and 25 µM

The number of HepG2 cells used was in the range of 5 × 10^5^ ~ 5 × 10^6^ cells/mL

Caspase-9 content was expressed as ng/mL

^*^Significantly different from the same concentration incubated with HepG2 cells for 48 h at *P* < 0.05, using independent Student’s *t *test

^a,b^Data in the same rows having the same superscript are significantly different from each other at *P* < 0.05 using one way analysis of variance (ANOVA) followed by Tukey as post hoc test

**Table 5:** Effects of ifosfamide on caspase-9 content in cell lysate of cultured HepG2 cells

***Time period***	***Control***	***Ifosfamide (µM)1***		
		**25**	**50**	**100**
**48 h**	4.0 ± 0.2^a^	12.1^a^ ± 0.7	20.3^a^ ± 1.4	30.9^a^ ± 3.1
**72 h**	4.2 ± 0.5^b^	15.0^*,b^ ± 1.3	28.1^*,b^ ± 1.9	40.75^*,b^ ± 2.3

Data represent mean ± SE, *n* = 3

1Ifosfamide was used in its IC50 value beside two of its fractions; 25 and 50 µM

The number of HepG2 cells used was in the range of 5 × 10^5^ ~ 5 × 10^6^ cells/mL

Caspase-9 content was expressed as ng/mL

^*^Significantly different from the same concentration incubated with HepG2 cells for 48 h at *P* < 0.05, using independent Student’s *t *test

^a,b^Data in the same rows having the same superscript are significantly different from each other at *P* < 0.05 using one-way analysis of variance (ANOVA) followed by Tukey as post hoc test

Notably, the cellular content of caspase-9 was elevated following exposure to glufosfamide more than ifosfamide. For instance, the IC_50_ value of glufosfamide (50 µM) exhibited cellular increases in the metalloproteinase contents amounted to 76 and 78% following exposure for 48 and 72 h, respectively, compared to the values observed for the IC_50_ of ifosfamide (100 µM) (Tables 4 and 5).

### Comparative effects of glufosfamide and ifosfamide on the expression of caspase-3 gene in HepG2 cells using RT-PCR

**Fig. 4:**
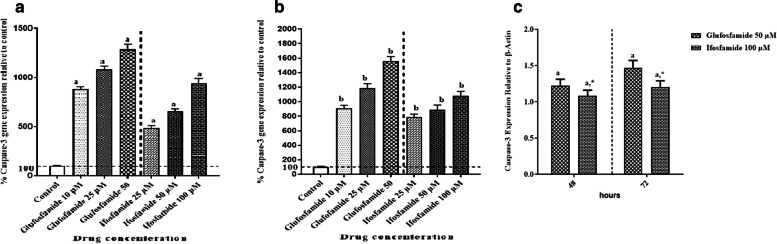
Effects of glufosfamide and ifosfamide on the expression of caspase-3 gene in HepG2 cells using RT-PCR. **a** Effects of glufosfamide or ifosfamide on the expression of caspase-3 gene in HepG2 cells at 48 h post-incubation using RT-PCR. **b** Effects of glufosfamide or ifosfamide on the expression of caspase-3 gene in HepG2 cells at 72 h post-incubation using RT-PCR. **c** Comparative effects of the IC50 of glufosfamide and ifosfamide on the expression of caspase-3 in HepG2 cells following exposure for 48 and 72 h

Glufosfamide upregulated the executioner gene; caspase-3 in a concentration-dependent manner. Exposure of HepG2 cells to glufosfamide at concentrations; 10, 25, and 50 µM for 48 h significantly induced expression of the effector caspase-3 amounted to more than sevenfold (778%), ninefold (978%), and 11-fold (1189%), respectively, compared to control untreated cells (Fig. 4a). Likewise, ifosfamide induced the expression of caspase-3 in a concentration-dependent. Herein, 48-h ifosfamide exposure at concentrations; 25, 50, and 100 µM upregulated the expression of caspase-3 by more than threefold (389%), fivefold (556%), and eightfold (844%), respectively, compared to untreated HepG2 cells (Fig. 4a).

Following 72-h exposure to glufosfamide at concentrations 10, 25, and 50 µM, there were increases in caspase-3 gene expression amounted to more than eightfold (811%), tenfold (1089%), and 14-fold (1456%), respectively, compared to untreated cells (Fig. 4b). Ifosfamide, on the other hand, upregulated the gene expression of caspase-3 in HepG2 cells following exposure to ifosfamide for 72 h at concentrations; 25, 50, and 100 µM by more than sixfold (689%), sevenfold (789%), and ninefold (978%), respectively, compared to control untreated cells (Fig. 4b).

It was apparent that 72-h exposure was optimal for the expression of caspase-3 compared to 48-h period. For instance the IC_50_ for glufosfamide (50 µM) significantly increased the gene expression following 72-h exposure by 21% compared to 48-h exposure time (Fig. 4c). However, the IC_50_ for ifosfamide (100 µM) at 72-h post-incubation had expression profile for caspase-3 gene that did not differ significantly from that following 48 h exposure (Fig. 4c).

### Comparative effects of glufosfamide and ifosfamide on the gene expression of Bcl2 in HepG2 cells

**Fig. 5:**
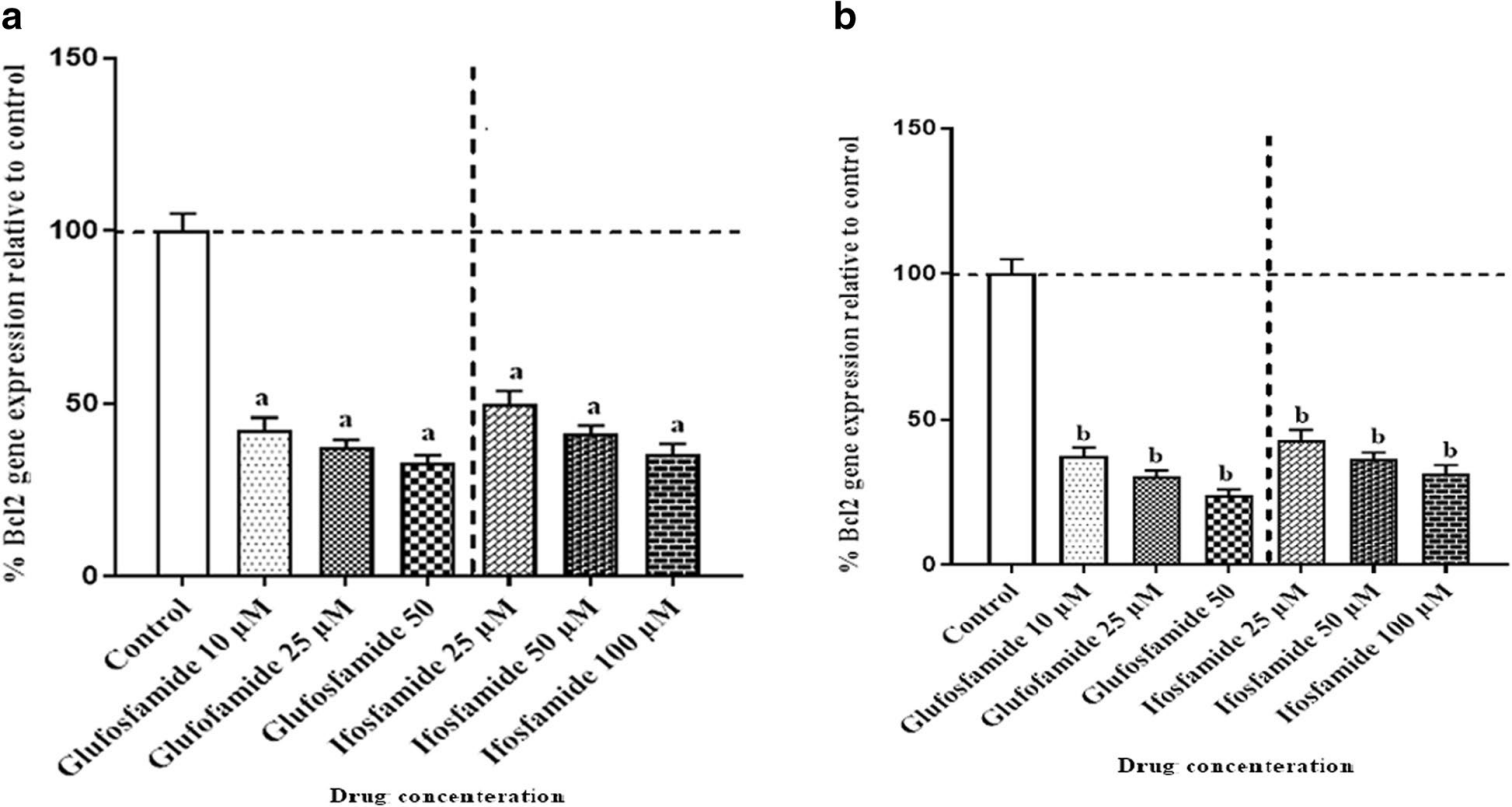
Effects of glufosfamide and ifosfamide on the gene expression of Bcl2 in HepG2 cells. **a** Effects of glufosfamide or ifosfamide on the expression of Bcl2 gene in HepG2 cells at 48 h post-incubation using RT-PCR. **b** Effects of glufosfamide or ifosfamide on the expression of Bcl2 gene in HepG2 cells at 72 h post-incubation using RT-PCR

Glufosfamide decreased the relative expression of the anti-apoptotic Bcl2 gene in HepG2 cells in a concentration-dependent manner. It decreased the expression of the gene by 57, 62, and 67% following exposure of HepG2 cells to concentrations; 10, 25, and 50 µM, respectively, for 48 h compared to untreated control cells (Table 6 and Fig. 5a). At 72-h post-incubation, the decreases in the gene expression were amounted to 62, 70, and 76% when glufosfamide was used at concentrations; 25, 50, and 100 µM, respectively, compared to untreated control cells (Table 6 and Fig. 5b). Upon incubation for 48 h, ifosfamide similarly decreased the expression of Bcl2 in HepG2 cells by 50, 59, and 65% when applied at concentrations; 25, 50, and 100 µM, respectively, compared to untreated cells (Table 7 and Fig. 5a). On the other hand, 72-h exposure to the same concentrations resulted in gene alterations amounted to 57, 64, and 69%, respectively, compared to control (Table 7 and Fig. 5b). On comparing the effects of either drug on Bcl2 expression at both time intervals, it was apparent that 72 h was the optimal exposure time. For instance, the IC_50_ of glufosfamide (50 µM) reduced the gene expression by 28% compared to the 48-h time period (Table 6). There was a slight nadir, however, in the expression of Bcl2 amounted to 12% compared to the 48-h interval when HepG2 cells were exposed to the IC_50_ of ifosfamide (100 µM) for 72 h (Table 7).

**Table 6:** Effects of glufosfamide on Bcl2 gene expression in HepG2 cells

***Time period***	***Control***	***Glufosfamide (µM)***^***1***^		
		**10**	**25**	**50**
**48 h**	2.4 ± 0.04	1.02^a^ ± 0.01	0.9^a^ ± 0.02	0.79^a^ ± 0.01
**72 h**		0.9^*,b^ ± 0.02	0.73^*,b^ ± 0.05	0.57^*,b^ ± 0.02

Data represent mean ± SE, *n* = 4

^1^Glufosfamide was used in its IC_50_ value beside two of its fractions; 10 and 25 µM

The number of HepG2 cells used was in the range of 5 × 10^5^ ~ 5 × 10^6^ cells/mL

Bcl2 content was normalized to the reference β-actin gene and expressed as percent expression relative to untreated control cells

^*^Significantly different from the same concentration incubated with HepG2 cells for 48 h at *P* < 0.05, using independent Student’s *t *test

^a,b^Data in the same rows having the same superscript are significantly different from each other at *P* < 0.05 using one-way analysis of variance (ANOVA) followed by Tukey as post hoc test

**Table 7:** Effects of ifosfamide on Bcl2 gene expression in HepG2 cells

***Time period***	***Control***	***Ifosfamide (µM)***^***1***^		
		**25**	**50**	**100**
**48 h**	2.4 ± 0.04	1.2^a^ ± 0.01	0.99^a^ ± 0.03	0.85^a^ ± 0.02
**72 h**		1.03^*,b^ ± 0.009	0.87^*,b^ ± 0.02	0.75^*,b^ ± 0.01

Data represent mean ± SE, *n* = 3

^1^Ifosfamide was used in its IC_50_ value beside two of its fractions; 25 and 50 µM

The number of HepG2 cells used was in the range of 5 × 10^5^ ~ 5 × 10^6^ cells/mL

Bcl2 content was normalized to the reference β-actin gene and expressed as percent expression relative to untreated control cells

^*^Significantly different from the same concentration incubated with HepG2 cells for 48 h at *P* < 0.05, using independent Student’s *t *test

^a,b^Data in the same rows having the same superscript are significantly different from each other at *P* < 0.05 using one-way analysis of variance (ANOVA) followed by Tukey as post hoc test

Glufosfamide used at its IC_50_ also significantly decreased Bcl2 expression by 24% when applied to culture medium for 72 h compared to the IC_50_ of ifosfamide (Tables 6 and 7). There was no significant difference between both glufosfamide and ifosfamide when applied to cultured HepG2 cells for 48 h at their respective IC_50_ values (Tables 6 and 7).

### Comparative effects of glufosfamide and ifosfamide on the mitochondrial membrane potential (Δψm) in HepG2 cells

**Fig. 6:**
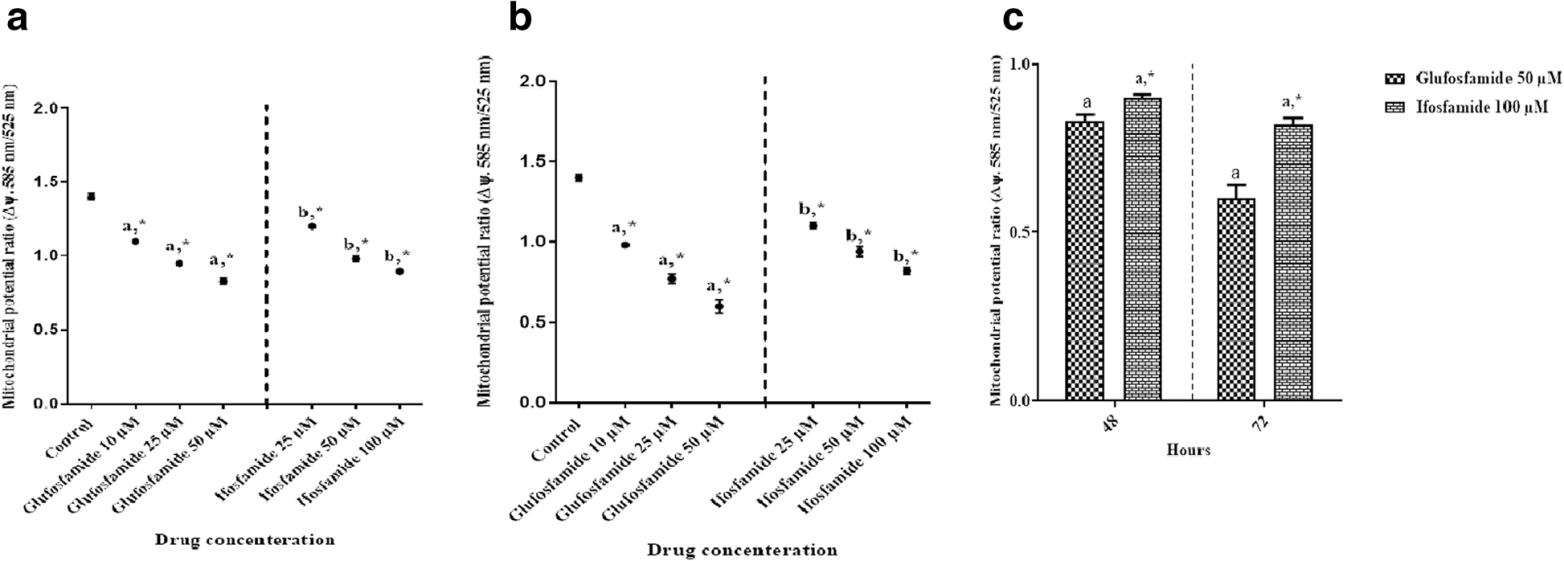
Effects of glufosfamide and ifosfamide on the mitochondrial membrane potential (Δψm) in HepG2 cells. **a** Effects of glufosfamide or ifosfamide on the mitochondrial membrane potential in HepG2 cells following exposure to 48 h. **b** Effects of glufosfamide or ifosfamide on the mitochondrial membrane potential in HepG2 cells following exposure to 72 h. **c** Comparative effects of the IC50 of glufosfamide and ifosfamide on the mitochondrial membrane potential in HepG2 cells following exposure for 48 and 72 h

Glufosfamide significantly lowered the mitochondrial membrane potential in HepG2 cells in a concentration-dependent manner following incubation for 48 h. There were significant decreases in Δψm amounted to about 21, 32, and 41% following exposure to the concentrations; 10, 25, and 50 µM, respectively, compared to untreated control cells (Fig. 6a). Similar results were observed following 72-h exposure. Glufosfamide used at the same concentration regimens significantly decreased Δψm by 30, 45, and 57%, respectively, compared to control cells (Fig. 6b). Likewise, ifosfamide deteriorated the mitochondrial membrane potential in a concentration-dependent manner at either 48 or 72 h. In this sense, following a 48-h exposure, the oxazaphosphorine drug lowered Δψm by about 14, 30, and 36%, respectively, when incubated with HepG2 cells at concentrations 25, 50, and 100 µM, respectively, compared to untreated control cells (Fig. 6a). The detriorations in Δψm values after a 72-h exposure were amounted to 21, 33, and 41% for the corresponding concentrations; 25, 50, and 100 µM, respectively, compared to control cells (Fig. 6b). It was obvious that mitochondrial membrane potential was much deteriorated following glufosfamide exposure for 72 h more than a 48-h exposure. For instance, the IC_50_ of glufosfamide lowered Δψm by 28% when the cells were incubated with the drug for 72 h compared to cells treated only for 48 h (Fig. 6c).

The same holds true for ifosfamide. Exposure of HepG2 cells to the IC_50_ (100 µM) resulted in mitochondrial potential nadir by 9% compared to the 48-h exposure period (Fig. 6c).

On comparing the effects of both drugs used at their respective IC_50_ values, glufosfamide significantly lowered the mitochondrial membrane potential by 8 and 27% following 48- and 72-h exposure, respectively, compared to ifosfamide-treated HepG2 cells (Fig. 6c).

### Comparative effects of glufosfamide and ifosfamide on ATP level in HepG2 cells

**Fig. 7:**
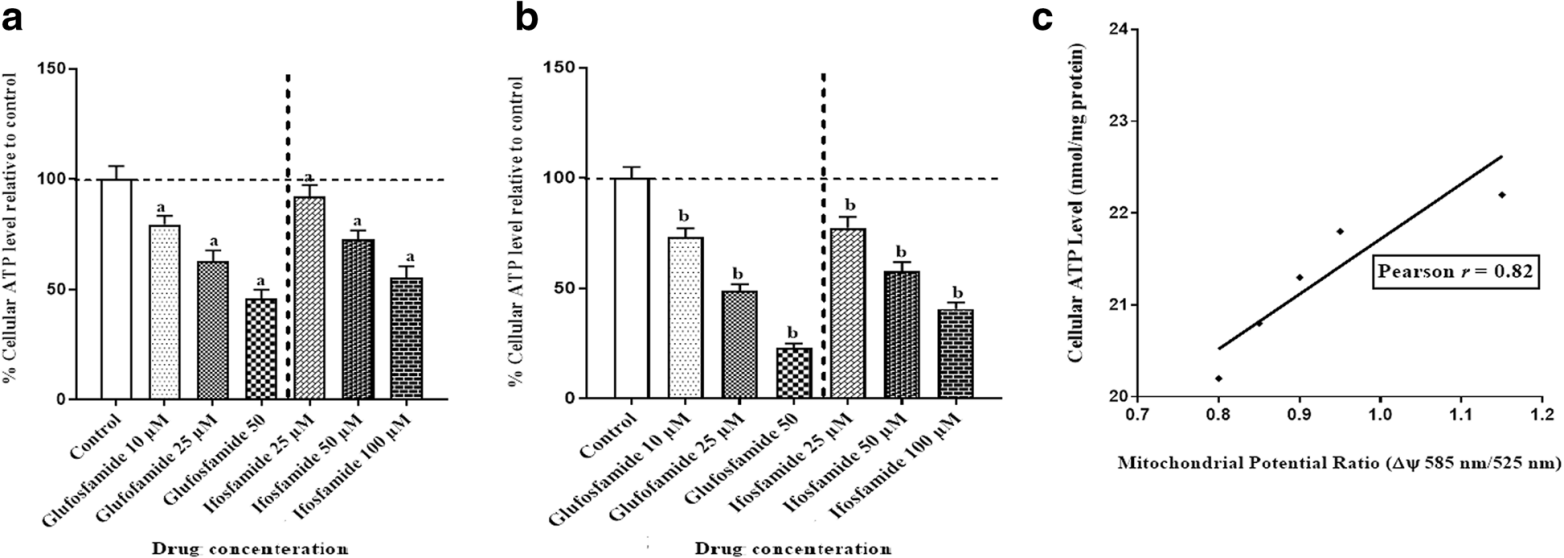
Effects of glufosfamide and ifosfamide on ATP level in HepG2 cells. **a** Effects of glufosfamide or ifosfamide on ATP level in HepG2 cells following incubation for 48 h. **b** Effects of glufosfamide or ifosfamide on ATP level in HepG2 cells following incubation for 72 h. **c** Correlation between mitochondrial membrane potential and ATP level in HepG2 cells

Glufosfamide altered ATP level in HepG2 cells in a concentration- and time-dependent manner. The cellular ATP levels were lowered by 21, 37, and 50% following exposure of HepG2 cells for 48 h to the concentrations: 10, 25, and 50 µM, respectively, compared to untreated control cells (Table 8 and Fig. 7a). At 72-h post-incubation, the same concentration regimens of glufosfamide exhibited notable decreases in cellular ATP levels amounted to 27, 51, and 67%, respectively; compared to control HepG2 cells (Table 8 and Fig. 7b). Likewise, ifosfamide reduced cellular ATP levels following exposure of HepG2 cells for 48 h at 50 and 100 µM by 23 and 44%, respectively, compared to non-treated control cells (Table 9 and Fig. 7a). The lowest concentration had, however, no effect on ATP level. Following exposure to the same concentrations, cellular alterations in ATP levels were amounted to 22, 42, and 59%, respectively, compared to non-treated HepG2 cells (Table 9 and Fig. 7b).

**Table 8:** Effects of glufosfamide on ATP level in HepG2 cells

***Time period***	***Control***	***Glufosfamide (µM)***^***1***^		
		**10**	**25**	**50**
**48 h**	20.9 ± 1.2	16.6^a^ ± 0.9	13.1^a^ ± 0.5	9.6^a^ ± 0.4
**72 h**		15.3^b^ ± 0.6	10.2^*,b^ ± 0.4	6.9^*,b^ ± 0.5

Data represent mean ± SE (*n* = 4)

ATP level is expressed as nmol/mg protein

^1^Glufosfamide was used in its IC_50_ value beside two of its fractions; 10 and 25 µM

The number of HepG2 cells used was in the range of 5 × 10^5^ ~ 5 × 10^6^ cells/mL

^*^Significantly different from the same concentration incubated with HepG2 cells for 48 h at *P* < 0.05, using independent Student’s *t *test

^a,b^Data in the same rows having the same superscript are significantly different from each other at *P* < 0.05 using one-way analysis of variance (ANOVA) followed by Tukey as post hoc test

**Table 9:** Effects of ifosfamide on ATP level in HepG2 cells

***Time period***	***Control***	***Ifosfamide (µM)1***		
		**25**	**50**	**100**
**48 h**	20.9 ± 1.2	19.3 ± 0.9	15.2^a^ ± 0.4	11.6^a^ ± 0.5
**72 h**		16.2^*,b^ ± 0.5	12.1^*,b^ ± 0.3	8.5^*,b^ ± 0.4

Data represent mean ± SE (*n* = 4)

ATP level is expressed as nmol/mg protein

^1^Ifosfamide was used in its IC_50_ value beside two of its fractions; 25 and 50 µM

The number of HepG2 cells used was in the range of 5 × 10^5^ ~ 5 × 10^6^ cells/mL

^*^Significantly different from the same concentration incubated with HepG2 cells for 48 h at *P* < 0.05, using independent Student’s *t *test

^a,b^Data in the same rows having the same superscript are significantly different from each other at *P* < 0.05 using one-way analysis of variance (ANOVA) followed by Tukey as post hoc test

There was also a notable deterioration in cellular ATP level following exposure to glufosfamide particularly at the IC_50_ (50 µM) for 72 h amounted to about 28% compared to the 48-h time period. Similarly, exposure of HepG2 cells to the IC_50_ of ifosfamide (100 µM) for 72 h resulted in a significant reduction in cellular ATP level amounted to 27% compared to 48-h exposure (Tables 8 and 9). Further, the cellular ATP levels following exposure to glufosfamide particularly the IC_50_ (50 µM) at 48- and 72-h interval were lowered by 17 and 19%, respectively, compared to ifosfamide-challenged cells at the corresponding time intervals (Tables 8 and 9). Interestingly, there was a positive correlation between mitochondrial membrane potential and cellular level of ATP in HepG2 cells with a Pearson correlation of *r*^2^ of about 0.82 (Fig. 7c).

## Discussion

The lack of tumor selectivity of anticancer drugs and the development of multidrug resistance have given impetus to the development of target-specific new cytotoxic compounds, in addition to modification of the targeted drug system to prolong the half-life of the therapeutic targeted system by conjugation as recently published [14].

Amongst the cancer drug targeting modalities recently developed is the concept of saccharides/anti-tumor conjugates; glycoconjugates, the anti-tumor drugs with specific glucose transporters, which transfer the drugs to the tumor cells, where they are selectively up taken and, thus, minimizing the organ toxicity of these compounds and maximizing their pharmacokinetic parameters, thereby reducing the administered doses of the glycodrugs [15].

The cytotoxicity of glufosfamide, let for its parent compound; ifosfamide in hepatocellular carcinoma has never been tested before. Taken together, the current study has been conducted in an attempt to address the possible cytotoxicity of glufosfamide in an in vitro liver tumor paradigm namely; HepG2 cells, in comparison to the parent compound; ifosfamide. Many facets of apoptosis pathway have been unraveled in the present study as a plausible mechanism for the oncolytic activity of both oxazaphosphorine drugs under investigation. The effects of both oxazaphosphorine drugs on mitochondrial membrane potential and consequent energy production have also been investigated.

Apparently, glufosfamide induced cytotoxicity in HepG2 cells in a concentration- and time-dependent manner with the optimal time for cell kill being at 72 h post-incubation. The same holds true for its aglycone; ifosfamide, yet the IC_50_ values were notably higher than glufosfamide. Indeed, these results are the first reported data for the cytotoxicity of any oxazaphosphorine drugs in HepG2 cells. The data is consistent with a study of other glucose conjugated drug adriamycin conjugated with 2-amino-2-deoxy-d-glucose and succinic acid (2DG–SUC–ADM) that induced a higher apoptosis than the free drug ADM [16]. The observed cytotoxicity in the current study is in line with previous reported preclinical data in other cancer cell lines [4, 17].

The mechanisms underlying glufosfamide cytotoxicity in the current study reside for the most part on the observed apoptotic effects. The tumor cell killing and apoptosis-inducing effects of glufosfamide as one of the oxazaphosphorines are resulted from DNA crosslink formation through covalent bonding of highly reactive alkyl groups of the alkylating nitrogen mustards, resulting from oxazaphosphorines with specific nucleophilic groups of DNA molecules. Oxazaphosphorine-induced apoptosis is always mediated by the mitochondrial pathway, leading to activation of the initiator caspase-9 which in turn activates the effector caspases-3 and caspase-7 [18]. In comparison to ifosfamide, the apoptotic effcts of glufosfamide were more profound and this is in harmony with their cytotoxic potentials. Herein, glufosfamide increased the positive annexin v-stained cells, increased caspase-9 cellular content, and upregulated the expression of caspase-3 gene and meanwhile downregulated Bcl2 gene expression.

Similar apoptotic effects were reported for glufosfamide and other oxazaphosphorines as addressed by various apoptotic assays such as the annexin v stain, caspases, and Bcl2. A strong decline in the level of Bcl-2 protein and activation of caspase-3, caspase-8, and caspase-9 were observed in an isogenic variant (CL-V5B) of the Chinese hamster V79 cells following incubation with glufosfamide. Apoptosis increased in a dose-dependent manner and was accompanied by induction of DNA double-strand breaks in the sensitive cells [17]. Another study determined that an enhancement of PC (prostate cancer) cells apoptosis by annexin v stain following glufosfamide treatment either in single or docetaxel combination drug treatment [19].

Indeed, the mechanisms of apoptosis are highly complex and sophisticated, involving an energy-dependent cascade of molecular events [20]. Irrespective of the exact mechanism involved, caspases are widely expressed in an inactive pro-enzyme form in most cells and once activated can often activate other pro-caspases, allowing initiation of a protease cascade. Some pro-caspases can also be aggregated and autoactivated. This proteolytic cascade, in which one caspase can activate other caspases, amplify the apoptotic signaling pathway, and thus lead to rapid cell death.

One of the biochemical features of apoptosis is the expression of cell surface markers that result in the early phagocytic recognition of apoptotic cells by adjacent cells, permitting quick phagocytosis with minimal compromise to the surrounding tissue. This is achieved by the movement of the normal inward-facing phosphatidylserine of the cell’s lipid bilayer to expression on the outer layers of the plasma membrane [21].

In the current study, caspase-9 was elevated in HepG2 cells following exposure to the oxazaphosphorine drugs. Likewise, caspase-3 was upregulated while Bcl2 was downregulated. In this sense, it is well known that once initiated, caspase-9 goes on to cleave procaspase-3 and procaspase-7, which cleave several cellular targets, including poly ADP ribose polymerase. Execution caspases activate cytoplasmic endonuclease, which degrades nuclear material, and proteases that degrade the nuclear and cytoskeletal proteins. Caspase-3, caspase-6, and caspase-7 function as effectors or “executioner” caspases, cleaving various substrates including cytokeratins, PARP, the plasma membrane cytoskeletal protein alpha fodrin, the nuclear protein NuMA, and others, that ultimately cause the morphological and biochemical changes seen in apoptotic cells [22].

Caspase-3 is considered to be the most important of the executioner caspases and is activated by any of the initiator caspases (caspase-8, caspase-9, or caspase-10). In brief, cytochrome c released during apoptosis binds to the adaptor molecule Apaf-1 and leads to the activation of caspase-9 through the formation of the multimeric apoptosome complex [23]. Caspase-9 then activates the central caspase in the apoptotic signaling machinery, caspase-3. Once caspase-3 is in action, apoptotic cell death is inevitable. The control and regulation of these apoptotic mitochondrial events occur through members of the Bcl-2 family of proteins [24]. It is thought that the main mechanism of action of the Bcl-2 family of proteins is the control of cytochrome c release from the mitochondria via regulation of mitochondrial membrane permeability. The Bcl-2 family of proteins governs mitochondrial membrane permeability and can be either pro-apoptotic or anti-apoptotic. Previous report indicated that Bcl-2 and Bcl-XL inhibit apoptotic death primarily by controlling the activation of caspase proteases [25].

To weld things together, one could speculate that glufosfamide and its parent compound; ifosfamide induced apoptosis in HepG2 cells through activation of the initiator caspase-9 that in turn activated the executioner caspase-3 that in association with the downregulation of the anti-apoptotic Bcl2 resulted in a cascade of apoptotic events that ultimately resulted in the observed programmed cell death in the current study. Of major interest was the finding in the present work that glufosfamide and ifosfamide deteriorated the mitochondrial membrane potential alongside with reduced cellular ATP production. It has been documented that maintenance of the mitochondrial membrane potential is critical for ATP synthesis [26].

An earlier study reported that chloroacetaldehyde, the metabolite of ifosfamide, collapsed the mitochondrial membrane potential of osteosarcoma Saos-2 cells, induced the release of cytochrome c from mitochondria to the cytosol, and significantly reduced cellular ATP levels during the course of death [27].

To explain the exact link between apoptosis and mitochondrial membrane potential, Ly et al. [28] adopted the following scenario. Early in apoptosis, the cells round up, losing contact with their neighbors and shrink. In the cytoplasm, the endoplasmic reticulum dilates and the cisternae swell to form vesicles and vacuoles. In the nucleus, chromatin condenses and aggregates into dense compact masses and is fragmented by endonucleases. The nucleus becomes convoluted and buds off into several fragments, which are encapsulated within the forming apoptotic bodies. In the plasma membrane, cell junctions are disintegrated, whereby the plasma membrane becomes active and convoluted, eventually blebbing. The cell breaks up in a florid manner leading to the “falling away” of several membrane spheres containing the “packaged” cellular contents identified as apoptotic bodies of various sizes.

It remains uncertain whether the loss of Δψm occurs as an initiator, or as an effect of apoptosis, or whether it is actually necessary for apoptosis induction to occur. Changes in Δψm have been originally postulated to be early and obligate events in the apoptotic signaling pathway. Multiple lines of research demonstrate that the nuclear features of apoptosis are preceded by changes in mitochondrial structure and Δψm in some regimes of induction of apoptosis [29]. Whether such Δψm deterioration is a first event prior to caspase activation or a sequel thereof, it is quite believed that caspase activation may provide a feedback amplification loop leading to dissipation of Δψm [28].

The previous report by Harris and Thompson (30) showed that Bcl-2 family proteins have been hypothesized to coordinate the permeability of both the outer and inner mitochondrial membranes through the permeability transition (PT) pore may lend support to this view. By regulating mitochondrial membrane physiology, Bcl-2 proteins also affect mitochondrial energy generation and thus influence cellular bioenergetics. Thereby, there was no wonder that by decreasing the mitochondrial membrane potential along with the downregulation of Bcl2 gene, glufosfamide, and its aglycone reduced ATP production in HepG2 cells. Hence, one could argue that the cytotoxicity of both oxazaphosphorine drugs observed in HepG2 cells may be ascribed to not just apoptotic signaling but presumably to alteration of energy production as well secondary to the deterioration of mitochondrial membrane potential.

## Conclusions

In conclusion, from the obtained data it is concluded that when well analyzed, the current study reported for the first time cytotoxicity activity of glufosfamide in hepatocellular carcinoma in vitro; namely, HepG2 cells relative to its parent aglycan ifosfamide. Glufosfamide showed a higher oncolytic activity in HepG2 cells than ifosfamide that was confirmed through MTT cytotoxic assay, flowcytometric analysis of apoptosis frequencies using Annexin V/PI double stain, and measurement of apoptotic markers (caspase-9, caspase-3, and Bcl2 gene expression) by immunocytochemical assay and real-time PCR. This in addition to shedding more light about the mechanism of apoptosis through measurement of mitochondrial membrane potential by the potentiometric dye; JC-1 and ATP production measured by the luciferase-chemiluminescence technique. The generated data warrants further elucidations by in vivo study.

## Data Availability

All data and materials are available upon request.

## Abbreviations

HCC: Hepatocellular carcinoma
HepG2: Human hepatoma cell line
IFO: Ifosfamide
GLU: Glufosfamide
Real time-PCR: Real-time polymerase chain reaction
LRU: Luminescence relative units
CPS: Count per second
ATP: Adenosine triphosphate
IC50: Half maximal inhibitory concentration
